# Global research trends and basis of venous/lymphatic malformations during 2003–2023: a bibliometric study over two decades

**DOI:** 10.3389/fmed.2025.1555168

**Published:** 2025-04-17

**Authors:** Hongyuan Liu, Jun Zhu, Hui Chen, Xiaoxi Lin

**Affiliations:** ^1^Department of Plastic and Reconstructive Surgery, Shanghai Ninth People’s Hospital, Shanghai Jiaotong University School of Medicine, Shanghai, China; ^2^Department of Urology, Shanghai Ninth People’s Hospital, Shanghai Jiaotong University School of Medicine, Shanghai, China

**Keywords:** bibliometric, venous malformation, lymphatic malformation, vascular malformation, vascular anomaly

## Abstract

**Introduction:**

Venous/lymphatic malformations (VM/LM) represent a group of vascular anomalies that share similarities in diagnosis, classification, management, and pathogenesis. Despite growing research interest, no comprehensive bibliometric analysis has been conducted to map the knowledge landscape and evolution of VM/LM studies.

**Methods:**

We searched the Web of Science Core Collection database for VM/LM-related publications from January 2003 to December 2023. multiple analytical tools including VOSviewer, CiteSpace, and R package ‘bibliometrix’ were used to analyze publication trends, collaboration networks, and research hotspots.

**Results:**

4,747 publications with 57,893 references were enrolled and analyzed. The number of publications is growing generally and erupted in 2019. The USA, China, and Japan are the top 3 countries in publication. The main study centers are scattered in the USA, Europe, China, and Japan. Fishman SJ published most. Vikkula M was cited most. Journal of Pediatric Surgery published and was cited most. The knowledge basis of VM/LM contains a wide range of concepts including classification/differential diagnosis, genetic causes, Sirolimus treatment, localized intravascular coagulopathy, and percutaneous treatment. The topic trend mainly changed after 2019 to genetic study and targeted therapy.

**Discussion:**

This is the first bibliometric analysis of VM/LM research demonstrating the field’s rapid growth and shifting research hotspots. Four key future directions emerged: molecular-level diagnosis and classification, precision targeted therapy, advanced sclerotherapy techniques, and artificial intelligence applications. International collaboration between countries and centers needs to be strengthen. These findings provide an objective foundation for future VM/LM investigations.

## Introduction

Venous malformation/lymphatic malformation (VM/LM) are a group of vascular malformations that share many similarities and commonalities in their diagnosis, classification, management, and pathogenesis. Symptoms of VM/LM vary from asymptomatic to severe or even life-threatening, depending on the subtypes, affected anatomic regions, and lesion sizes. VM/LM is traditionally diagnosed through disease course, clinical examination, imaging, and pathology. In recent years, genetic sequencing has emerged as a useful tool in VM/LM diagnosis. Treatment of VM/LM includes surgical excision, sclerotherapy, administration of agents to control symptoms, and targeted therapy (1–4).

Despite numerous attempts in basic and translational research and clinical innovations, extensive and complex VM/LM remains incurable and is difficult to manage (5, 6). Surgery is suitable only for local debulking or localized lesions, whereas sclerotherapy has limitations in treating extensive lesions or those in special regions, which may lead to refractory or severe adverse events (7). Targeted therapy has been beneficial for VM/LM patients. However, sirolimus has shown effectiveness only in controlling symptoms reducing limited lesions, rather than completely eliminating all lesions (8). In addition, the pathogenesis of VM/LM is not well clarified, especially for non-mutated cases.

Bibliometrics is a literature analysis approach to explore the knowledge map and publication characteristics quantitatively and qualitatively using mathematical, statistical, and other econometric methods (9). It provides a comprehensive understanding of relationships among authors, institutions, countries, journals, and references in certain research fields. Bibliometrics can not only determine the existing trend but also identify further scope and inspire future studies.

This work presents the first comprehensive bibliometric analysis of the current situation of global VM/LM studies over the period of 2003–2023 based on data obtained from the Web of Science Core Collection (WoSCC) database, aiming to provide insights into this field and predictions for future studies.

## Methods

### Data sources and search strategy

**Figure 1:**
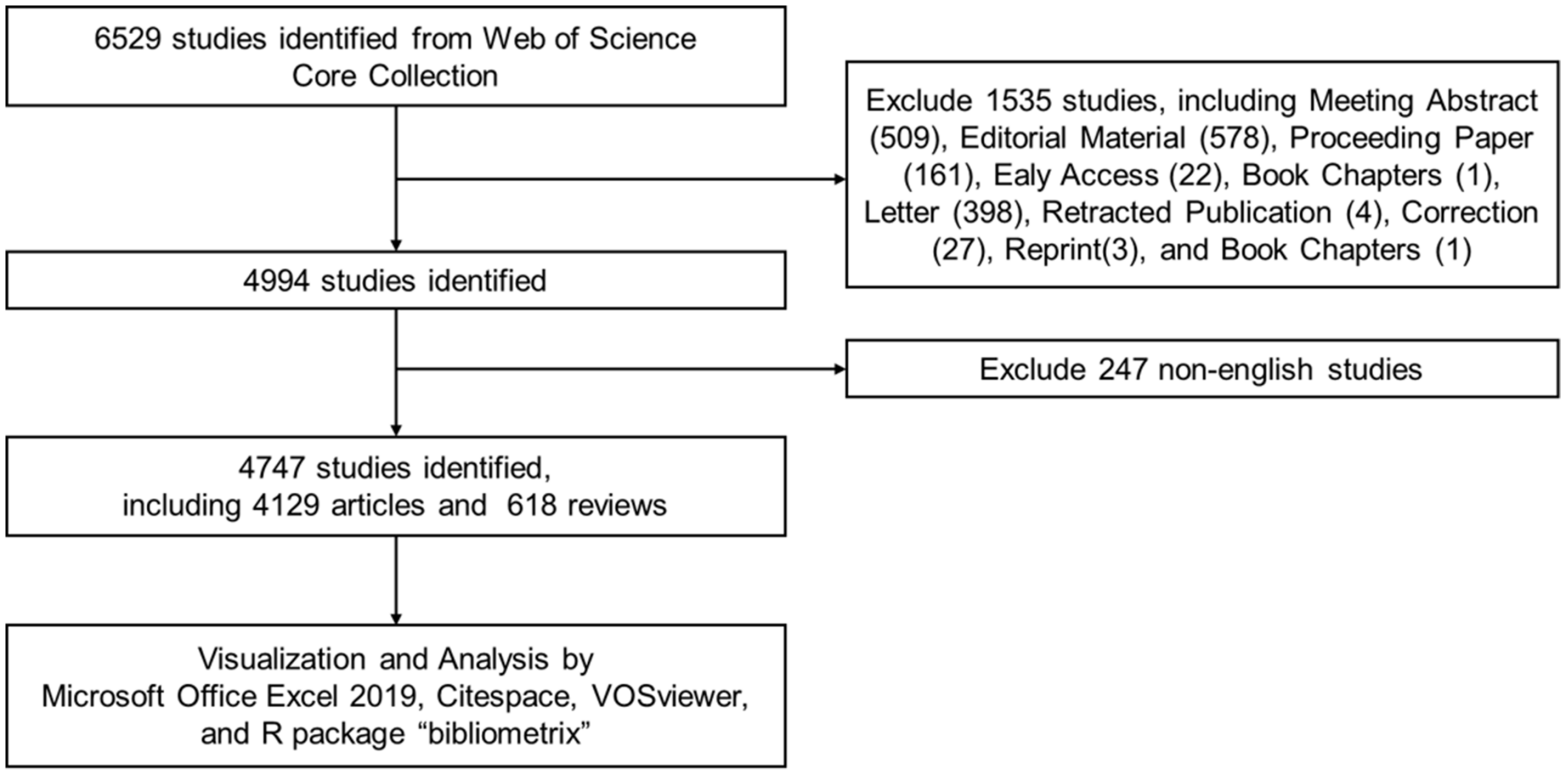
Workflow of the data search strategy.

All data were searched and retrieved from the WoSCC database^1^, covering the period from 1 January 2003 to 31 December 2023. The search term consists of two parts: one is venous/lymphatic malformations (search term is #1, TS = (“venous malformation*” OR “Familial venous malformation cutaneous-mucosal” OR “Blue rubber bleb nevus syndrome” OR “Bean syndrome” OR “Glomuvenous malformation*” OR “Familial intraosseous vascular malformation” OR “Verrucous venous malformation*” OR “verrucous hemangioma*” OR “lymphatic malformation*” OR “lymphatic anomal*” OR “Kaposiform lymphangiomatosis” OR “Gorham-Stout disease” OR “lymphangioma*” OR “lymphovenous malformation*” OR “lymphatic-venous malformation*”)) and the other is the exclusion of cerebral-related studies (search term is #2, TS = (“Cerebral*” OR “brain” OR “Intracranial” OR “Central Nervous System”)). The final search term is “#1 NOT #2 AND PY = 2003–2023.” A total of 4,747 studies met our search criteria. Inclusion and exclusion criteria are shown in detail in Figure 1.

### Bibliometric analysis

The prediction model f (x) = ax^3^ + bx^2^ + cx + d was generated using Microsoft Excel 2019 to analyze past publication trends and future patterns, in which f (x) represents the total number of publications over a specific year and x represents time. The H-index (a scholar or a region has published H papers that have each been cited at least H times) was calculated to assess the productivity and citation impact of a scholar or a region (10). VOSviewer (version 1.6.20) was used to analyze relationships between authors, countries, institutions, journals, references, and keywords. CiteSpace (version 6.2.R4) was used to map the journals and analyze references. Other analyses, including annual production by each country, H-index, authors who contributed the most, journals, and references, were performed using the R package “bibliometrix” (version 4.2.3).^2^ The quartile and impact factor of the journals were obtained from the latest version of Journal Citation Reports. All quantitative analysis of publications was carried out using Microsoft Office Excel 2019, and the exact functions and parameters used in the aforementioned tools are provided in Supplementary file.

## Results

### Overview of publications on venous/lymphatic malformations

In accordance with the previously mentioned search strategy, 4,747 articles published from 2003 to 2023 were analyzed in this study, including 4,129 articles and 618 reviews. The workflow of the screening procedure is shown in Figure 1. The total number of references was 57,893, and the average citation per document was 15.09. The primary information on VM/LM publications is provided in Table 1.

**Table 1:** General information on VM/LM publications from 2003 to 2023.

Description	Results
Main information	
Timespan	2003:2023
Sources (journals, books, etc.)	1,098
Documents	4,747
Average citations per document	15.09
References	57,893
Keyword	
Keywords Plus (ID)	4,641
Author	
Authors	19,096
Co-authors per document	5.58
International co-authorships %	11.4
Document types	
Article	3,973
Article; book chapter	1
Article; early access	15
Article; proceedings paper	138
Article; retracted publication	3
Review	614
Review; early access	3

### Trends in annual research publication quantity

**Figure 2:**
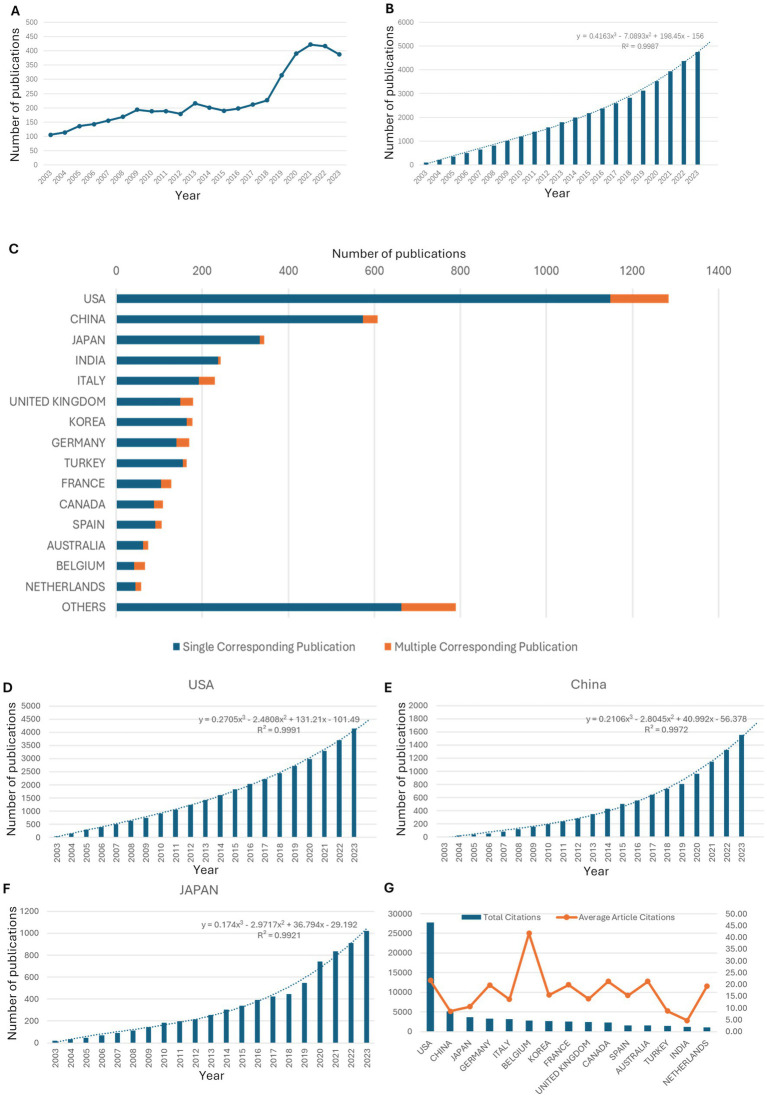
Trends in the number of publications. **(A)** The annual number of global new publications. **(B)** The cumulative number of global publications. The formula of the prediction model and R2 was shown. **(C)** Publications with respect to the corresponding authors’ countries. **(D)** The cumulative number of publications produced by the USA. The formula of the prediction model and R2 was shown. **(E)** The cumulative number of publications produced by China. The formula of the prediction model and R2 was shown. **(F)** The cumulative number of publications produced by Japan. The formula of the prediction model and R2 was shown. **(G)** The total number of citations and average number of article citations by the top 15 countries.

The number of new publications on VM/LM every year is shown in Figure 2A. Overall, the number of publications increased slightly every year from 2003 to 2010, remained stable from 2011 to 2018, and reached its peak during 2019. According to a model based on the curves of cumulative numbers of publications (Figure 2B), the number of publications will continue to grow steadily.

### Countries’ contributions to global publications

Over the past two decades, based on the country of the corresponding author of the publications, the USA produced the highest number of publications (1,284, 27%), followed by China (607, 12.8%) and Japan (344, 7.2%) (Figure 2C). With respect to the total number of publications so far, the USA, China, and Japan contributed the most, which fits the growth model as global trends (Figures 2D–F). Notably, the USA and China showed steady growth in new publications (Supplementary Figure 1A). Regarding publication time, the USA, Japan, and Germany were the primary contributors around 2016, whereas China and India were the primary contributors around 2018. Furthermore, regarding citations, the USA was the most cited country, well ahead of others, and China was the second, followed by Japan, Germany, and Italy (Figure 2G).

### Institutional distribution of publications

**Figure 3:**
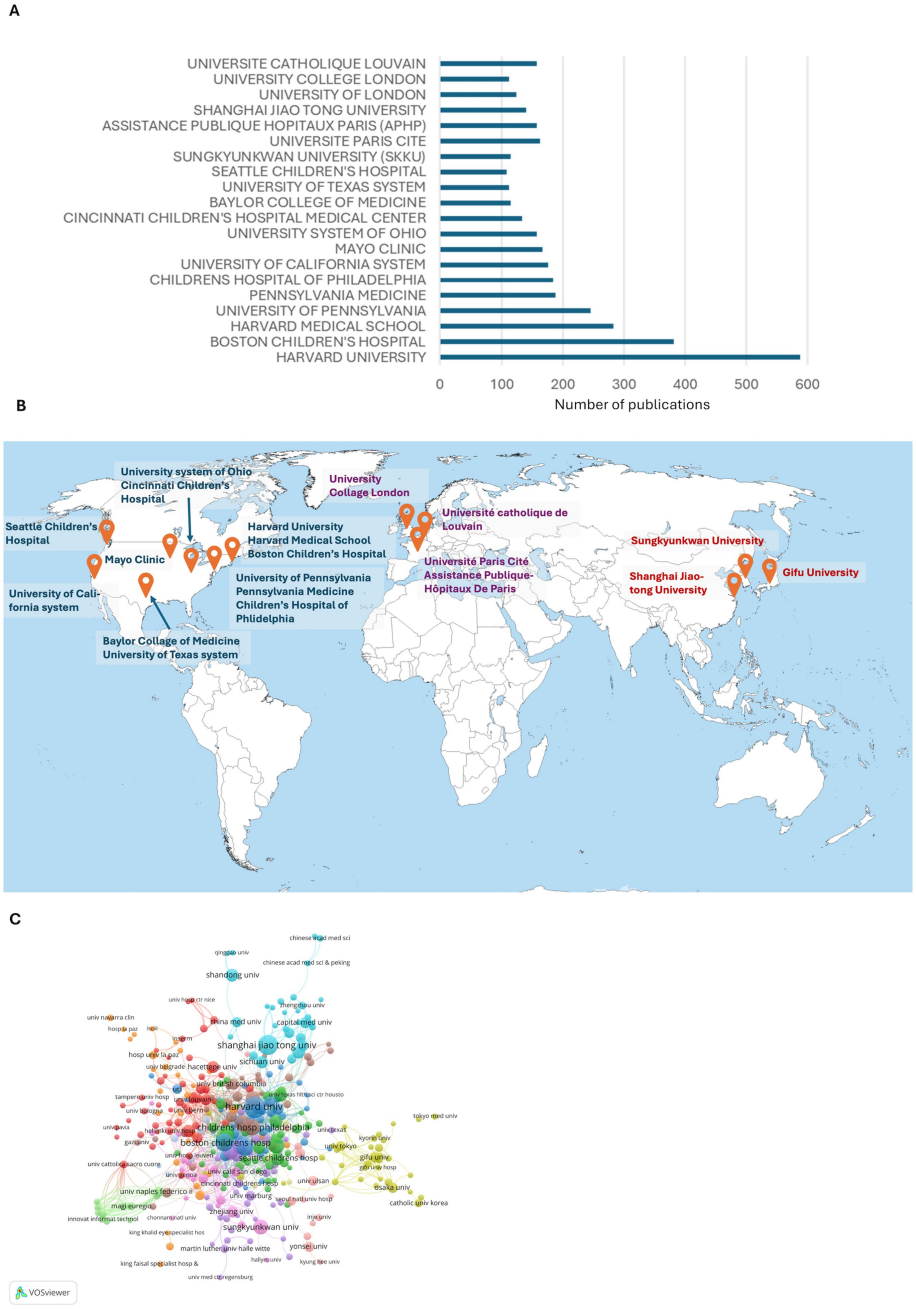
Distribution of institutions with the highest number of publications in VM/LM research. **(A)** The number of publications of the 20 institutions with the highest number of publications. **(B)** The world map of major VM/LM study centers. Antarctica is hidden. **(C)** The collaborative network of institutions.

Harvard University, Boston Children’s Hospital, and Harvard Medical School published the highest number of studies. Among the 20 institutions with the highest number of publications, 13 were from the USA, 2 from France, 2 from Britain, and 1 each from Belgium, China, and Korea (Figures 3A,B).

While analyzing the collaborative network in each institution, 13 clusters were identified (Figure 3C). Harvard University emerged as the leading institution connected strongly with related institutions such as Boston Children’s Hospital, and other main institutions such as the University of Pennsylvania (and related institutions), Cincinnati Children’s Hospital Medical Center, and Seattle Children’s Hospital, thus becoming the VM/LM study center in the USA. Other leading study centers were University of Catholique Louvain, Universite Paris Cite, and other European institutions. Gifu University and other Japanese/Korean institutions, was the leading study center in East Asia, and Shanghai Jiao Tong University in China was another important study center in East Asia along with other Chinese institutions.

### Author distribution of publications

Consistent with Lotka’s law, less than 0.1% of authors published six or more than six articles (Supplementary Figure 2). As shown in Table 2, 19 authors published more than 20 publications. SJ Fishman from Boston Children’s Hospital made the highest number of publications on VM/LM, with 55 publications. M Vikkula from Universite Catholique Louvain was the most cited author, with an H-index of 26.

**Table 2:** The list of authors who published more than 20 publications.

Authors	Articles	H-index	Publication year started
Fishman SJ	55	24	2003
Mulliken JB	44	27	2004
Vikkula M	39	26	2004
Perkins JA	38	20	2004
Alomari AI	35	20	2006
Boon LM	34	24	2004
Greene AK	29	17	2007
Lee BB	29	24	2003
Adams DM	27	16	2011
Richter GT	27	11	2007
Ozeki M	26	13	2007
Zhao YF	25	11	2004
Wang L	24	10	2008
Wohlgemuth WA	23	10	2016
Werner JA	22	12	2008
Chen H	21	8	2009
Eivazi B	21	13	2008
Lin XX	21	8	2008
Van Der Horst CMAM	21	13	2003

**Figure 4:**
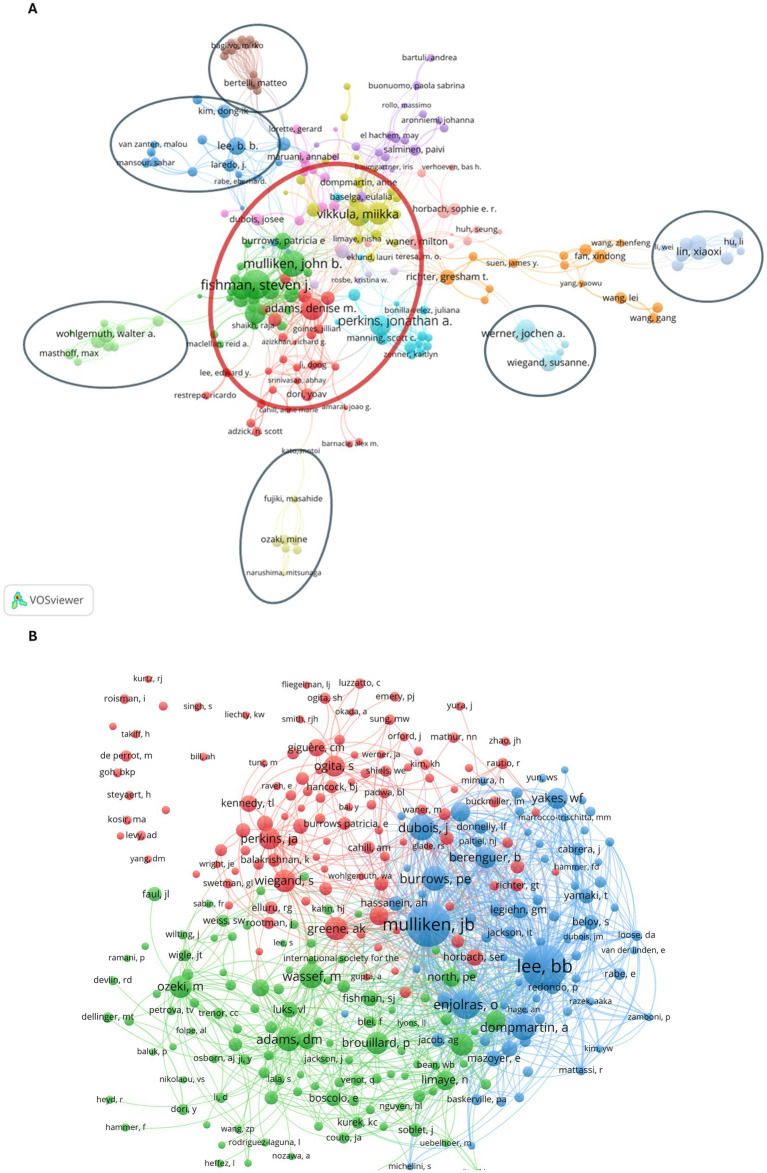
Network of the authors. **(A)** The collaboration network of authors. The red circle shows the core author groups. Blue circles show the independent author groups. **(B)** The co-citation network of authors.

In addition, we analyzed the collaboration network and found 15 clusters (Figure 4A). Boon LM and Vikkula M from Universite Catholique Louvain; Fishman SJ, Mulliken JB, and Alomari AI from Boston Children’s Hospital; Adams DM and Dori Y from Children’s Hospital of Philadelphia; and Perkins JA from Seattle Children’s Hospital were the leading authors of core author groups that were closely connected (red circle in Figure 4A). Interestingly, Lee BB from George Washington University Medical Center; Lin XX from Shanghai Jiao Tong University; Ozaki M from Kyorin University School of Medicine; Bertelli M from MAGI’s Lab, Italy; Werner JA from Philipps University, Marburg; and Wohlgemuth WA from University Medical Center Regensburg were the leading authors of the independent cluster connected indirectly to the core group.

We identified 299 co-cited authors who were cited more than 40 times, and three clusters (Figure 4B). Mulikken JB, Enjolras O, and Lee BB had active connections with multiple authors in the same cluster. Adams DM, Ozeki M, and Boon LM had connections together.

### Journal distribution of VM/LM

Following Bradford’s law, we identified 48 journals in the first zone that constituted core journals in this field (Supplementary Table 1). The 20 most relevant journals, the 20 most locally cited journals, and their local H-index and IF are presented in Tables 3, 4. The Journal of Pediatric Surgery had the highest number of publications and citations, with an H-index of 23, which is the highest among journals. Three other journals with a high number of publications were the Journal of Craniofacial Surgery, International Journal of Pediatric Otorhinolaryngology, and Phlebology. Three highly cited journals were the Plastic and Reconstructive Surgery, American Journal of Roentgenology, and Radiology. Furthermore, high-impact journals, such as the New England Journal of Medicine (two publications), Nature (one), Lancet (one), and Journal of the American Medical Association (one) published articles on VM/LM.

**Table 3:** The 20 most relevant journals.

Journal name	Articles	H-index	JCR category quartile	IF 2023
Journal of Pediatric Surgery	92	23	Q1	2.4
Journal of Craniofacial Surgery	83	12	Q3	1
International Journal of Pediatric Otorhinolaryngology	77	20	Q3	1.2
Phlebology	70	20	Q3	1.6
Pediatric Dermatology	55	13	Q3	1.2
Cureus Journal of Medical Science	54	4	Q3	1
International Journal of Surgery Case Reports	48	6	Q4	0.6
Journal of Pediatric Surgery Case Reports	48	2	Q4	0.2
Journal of Vascular Surgery-Venous And Lymphatic Disorders	47	11	Q1	2.8
Ophthalmic Plastic and Reconstructive Surgery	47	13	Q3	1.2
Pediatric Radiology	47	16	Q2	2.1
Medicine	43	11	Q2	1.3
Pediatric Blood & Cancer	42	17	Q1	2.4
Dermatologic Surgery	40	13	Q1	2.5
Laryngoscope	38	2	Q1	2.2
Lymphatic Research and Biology	38	10	Q3	1.6
BMJ Case Reports	37	3	Q3	0.6
Cardiovascular and Interventional Radiology	37	14	Q2	2.8
Journal of Vascular and Interventional Radiology	37	14	Q2	2.6
American Journal of Roentgenology	35	19	Q1	4.7

**Table 4:** The 20 most cited journals.

Journal name	Citied times	H-index	JCR Category quartile	IF 2023
J Pediatr Surg	2,911	23	Q1	2.4
Plast Reconstr Surg	2,538	12	Q1	3.2
Am J Roentgenol	2091	19	Q1	4.7
Radiology	1893	7	Q1	12.1
J Am Acad Dermatol	1,467	19	Q1	12.8
J Vasc Surg	1,429	19	Q1	3.9
Radiographics	1,400	23	Q1	5.2
Pediatrics	1,345	13	Q1	6.2
Arch Dermatol (JAMA Dermatology)	1,299	9	Q1	11.5
Pediatr Radiol	1,251	16	Q2	2.1
J Vasc Interv Radiol	1,229	14	Q2	2.6
Int J Pediatr Otorhi	1,126	20	Q3	1.2
Otolaryng Head Neck	1,085	18	Q1	2.6
Laryngoscope	1,007	15	Q1	2.2
Dermatol Surg	971	13	Q1	2.5
Arch Otolaryngol (JAMA Otolaryngology–Head & Neck Surgery)	961	11	Q1	6
Phlebology	928	20	Q3	1.6
Pediatr Blood Cancer	880	17	Q1	2.4
Am J Surg Pathol	847	11	Q1	4.5
Pediatr Dermatol	841	13	Q3	1.2

Journal network analysis revealed three major clusters (Supplementary Figure 3). Cluster 1 consisted of pediatrics and dermatological journals. Cluster 2 primarily comprised craniofacial, maxillofacial, and oral journals. Cluster 3 featured clinical vascular journals. In addition, cluster 4 consisted of basic science studies.

### Analysis of citations and references in VM/LM research

The 10 most locally cited publications are presented in Table 5, encompassing a wide range of VM/LM concepts, including classification, differential diagnosis, genetic causes, sirolimus treatment, localized intravascular coagulopathy, and percutaneous treatment. The 10 most cited references are presented in Table 6. Notab,y, five articles are unique compared with the 10 most locally cited publications. The studies published between 2000 and 2016 had the highest number of references.

**Table 5:** The 10 most locally cited publications.

Document	DOI	Year	Local citations (LC)	Global citations (GC)	LC/GC ratio (%)
Wassef et al. (24)	10.1542/peds.2014-3673	2015	319	798	39.97
Hammill et al. (25)	10.1002/pbc.23124	2011	186	411	45.26
Luks et al. (26)	10.1016/j.jpeds.2014.12.069	2015	151	335	45.07
Burrows et al. (29)	10.1097/01.RVI.0000124949.24134.CF	2004	139	210	66.19
Dompmartin (39)	10.1001/archderm.144.7.873	2008	138	204	67.65
Lee et al. (13)	10.1067/mva.2003.91	2003	129	209	61.72
Limaye (40)	10.1038/ng.272	2009	125	297	42.09
Dompmartin et al. (33)	10.1258/phleb.2009.009041	2010	116	181	64.09
Puig (41)	10.1007/s00247-002-0838-9	2003	110	144	76.39
Perkins (32)	10.1016/j.otohns.2010.02.026	2010	106	147	72.11

**Table 6:** The 10 most cited references.

Cited references	DOI	Citations
Mulliken et al. (11)	10.1097/00006534-198203000-00002	540
Wassef et al. (24)	10.1542/PEDS.2014-3673	319
Adams et al. (15)	10.1542/PEDS.2015-3257	232
Berenguer et al. (28)	NA; PMID:10597669	211
Hammill et al. (25)	10.1002/PBC.23124	186
Luks et al. (26)	10.1016/J.JPEDS.2014.12.069	151
Alqahtani et al. (30)	10.1016/S0022-3468(99)90590-0	149
Deserres (31)	10.1001/archotol.1995.01890050065012	142
Burrows et al. (29)	10.1097/01.RVI.0000124949.24134.CF	139
Dompmartin (39)	10.1001/ARCHDERM.144.7.873	138

The co-cited references network map was constructed using 385 references with more than 30 co-citations (Supplementary Figure 4), and three clusters were identified. Cluster 1 included 158 references, with Mulliken JB, 1982, Plastic Reconstructive Surgery, having the highest number of citations. Cluster 2 included 148 references, with Wassef M, 2015, Pediatrics, and Adams DM, 2016, Pediatrics, having the highest number of citations, respectively. Cluster 3 included 79 other references.

### Keywords and trending hotspots in VM/LM

**Figure 5:**
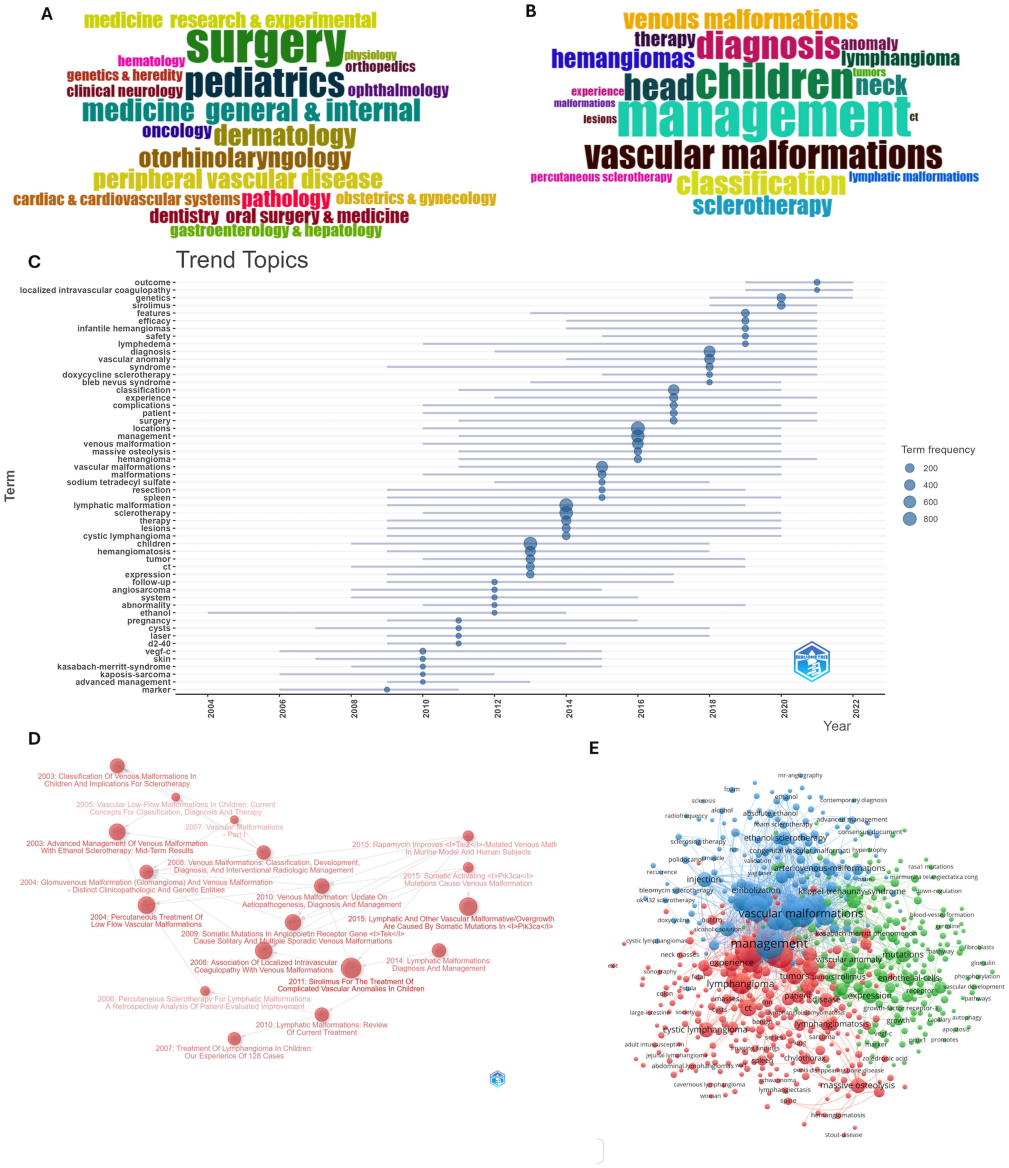
Keywords, topics, and historiography of VM/LM research. **(A)** Word cloud of the 20 most frequent WoS subject categories. **(B)** Word cloud of the 20 most frequent keywords. **(C)** Trend topics over the years. **(D)** Historiography of publications. **(E)** Network of keywords.

According to WoS subject categories, the majority of VM/LM studies belong to surgery, pediatrics, and radiology nuclear medicine and medical imaging (Figure 5A). The 20 most frequent keywords are shown in Figure 5B. The trend topics over the years are shown in Figure 5B. Prior to 2019, topics primarily centered on terminology, classifications, and diagnosis of vascular malformation, as well as their features, locations, and sclerotherapy. After 2019, research topics have shifted toward genetics, sirolimus treatment, and localized intravascular coagulopathy. The historiography of VM/LM, elucidated using 18 publications, is represented in Figure 5C, encompassing classification, diagnosis, management, and pathogenesis.

We clustered the Keywords Plus occurred more than 5 times into 3 main clusters (Figures 5D,E). Cluster 1 had 276 keywords that were primarily referring to diagnosis, classification, and management of VM/LM. Cluster 2 included 218 keywords that mainly focused on sclerotherapy. Cluster 3 included 160 items that were primarily related to genetic study and targeted therapy.

## Discussion

### General information on VM/LM research

Over the past 20 years, the number of publications on VM/LM has gradually increased. The USA has contributed the highest number of relevant studies, followed by China and Japan. During this rapid growth phase, the number of new publications from the USA and China has shown a steady increase over the years. Traditionally, the USA, Japan, and Germany are considered advanced countries regarding VM/LM research, while China and India are considered emerging contributors. The highest number of leading institutions are primarily located in the USA, France, Britain, Belgium, China, and Korea. This observation reflects the scientific and economic strength of the developed countries and their early start in this field. It also implies that VM/LM research has gained worldwide attention, with contributions from numerous other countries increasing over time.

However, it is difficult to determine the main cluster-by-country relationship despite their strong associations (Supplementary Figure 1B). This indicates extensive international cooperation between various countries focusing on this relatively rare condition.

The distribution of authors generally aligns with the study centers but appears to be more subdivided. The authors in the USA study center included multiple factions. The most published authors Fishman SJ and Mulliken JB focused on the diagnosis, classification, and management of VM/LM (11, 12). However, Lee BB and Alomari AI focused on sclerotherapy (13, 14). Furthermore, Adams DM focused on targeted therapy (15). The authors in European study centers focused on basic science study. Vikkula M and Boon LM focused on genetic pathogenesis in VM/LM (16). Moreover, Guillaume C focused on vascular malformation studies combined with basic science and clinical trials (17, 18). In addition, top authors in China and Japan such as Lin XX and Ozeki M published articles on a wide range of concepts (6, 19–23).

Regarding journals, the Journal of Pediatric Surgery had the highest number of both publications and citations, while other journals showed inconsistent patterns between the number of publications and citations. According to the journal network analysis, pediatric, dermatologic, oral surgical, and perivascular surgical journals have shown interest in VM/LM research. Several basic science journals have also published related studies.

### Knowledge base of VM/LM

The 10 most locally cited references or publications were selected to clarify the research basis of VM/LM. The endothelial-characteristic-based classification published by Mulliken JB in 1982, later adopted by international society for the study of vascular anomalies (ISSVA) classification, formed the foundation of the modern classification of vascular malformations with the highest number of citations (11, 24). Adams DM and Hammill AM established the sirolimus treatment base (15, 25), whereas Luks VL and Limaye N provided evidence on genetic pathogenesis (26, 27). Berenguer B, Burrows PE, Puig S, and Lee BB strengthened the research on sclerotherapy of VM/LM (13, 28, 29). Alqahtani A, Deserres LM, and Perkins JA provided detailed knowledge on LM (30–32). Dompmartin A introduced the concept of localized intravascular coagulation (LIC) and summarized VM knowledge base before 2010 (33). According to the reference spectroscopy, most knowledge base of VM/LM was established during 1999–2018, except for those from 1982 when Mulliken JB set the standard of VM diagnostics.

### Focuses and hotspots in VM/LM

The growth of VM/LM studies can be divided into three phases, reflecting shifts in research focus: slow growth phase (2003–2010), stable phase (2011–2018), and rapid growth phase (2019-present). In the slow growth phase, researchers mainly emphasized (a) diagnosis, classification, and terminology; (b) sclerotherapy; (c) coagulation disorders; and (d) pathogenesis. Several basic concepts and terms were investigated and established. Sclerotherapy was widely applied in VM/LM as a novel non-surgical treatment. In the stable phase, researchers consolidated previously established concepts and identified more subtypes. Special types associated with VM/LM were found. Studies on genetic therapy and sirolimus treatment gradually gained popularity. Of note, the ISSVA classification of vascular anomalies was widely accepted at this time, and studies focusing on genetic pathogenesis and sirolimus treatment had increased in 2015. In the rapid growth phase, genetic study and targeted therapy became the focus. As multiple mutations driven by numerous factors were found, various targeted drugs were studied, with sirolimus receiving the highest level of attention.

We identified four future research directions for VM/LM.

The first direction is diagnosis, classification, and management at the molecular level. Identification of genetic factors (and the molecules encoded by these genetic factors) that intervene in different signaling pathways will allow the development of targeted therapies in the future, including gene therapy (34). Classification and terminology of different VM/LM subtypes reflect the growing understanding achieved through investigations into the historiography of VM/LM research. With the increase in genetic findings (34), more syndromes have been recognized, such as PIK3CA-related overgrowth spectrum (PROS), Gorham–Stout disease, and lymphatic anomalies. Given the complexity of VM/LM and related syndromes, a large proportion of patients still lack a clear genetic diagnosis and need to be classified at the molecular level, encompassing more than just genetic factors.

The second direction in VM/LM research is precision and personalized targeted therapy. Sirolimus and other new emerging agents such as alpelisib (17) and trametinib (35) are beneficial to patients with extensive and complex VM/LM, generating numerous publications. Despite multiple pieces of high-level evidence supporting the efficacy of sirolimus and alpelisib (36), specific indications remain vague. We need high-quality clinical studies to obtain answers to the following questions: (a) Should those patients without mutations take targeted agents? (b) In which condition should patients start and stop targeted therapy? (c) What are the differences in efficacy between various agents?

The third direction in VM/LM research is sclerotherapy. The role of surgery and sclerotherapy as the first-line treatment for VM/LM remains uncertain. Sclerotherapy is improving every day in terms of new agents and methods. Regarding agents, alcohol, foam, and bleomycin have become classic; the iteration and combination of them are considered old concepts. Extracellular vesicles, targeted agents, hydrogels, and other advanced materials are emerging hotspots. Clinical studies should compare these novel materials or agents with the classic agents to ensure their efficacy and safety. With respect to methods, sclerotherapy can be image-guided using ultrasound, CT, and MRI from published studies. A few new studies have focused on how augmented reality (AR) and virtual reality (VR) can be used in sclerotherapy procedures. AR and VR can be applied in pre-surgery design, guidance during surgery, and efficacy evaluation after surgery. They may enable robot-assisted surgery and remote surgery.

The fourth research direction is the application of artificial intelligence (AI) in multiple fields of VM/LM. With the rapid development and increase in public use, the AI trend in the scientific and medical fields is inevitable. However, few VM/LM studies have focused on AI to date, according to our analysis. Mastering AI is important, urgent, and profitable. Several attempts have been made to use AI in automated diagnosis, differential diagnosis, lesion analysis, and surgery assistance (37).

### Advantages and limitations

To our knowledge, this is the first bibliometric study of VM/LM. Using multiple bibliometric software, we objectively presented the comprehensive situation of VM/LM research over the past 20 years and suggested perspectives for future investigations. In addition, there are several limitations to be mentioned. First, only articles and reviews in English from the WoSCC database were included, which did not encompass all publications. Second, the large volume of articles prevented the screening of studies. This might have caused the inclusion of irrelevant publications, which could have interfered with the results. Finally, our study has an inherent time lag due to its retrospective nature. Some cutting-edge technologies that are only presented in conferences, such as organoids (38), new drugs, and AI, are not included in the review.

## Conclusion

VM/LM comprises relatively rare vascular malformations that share similarities in clinical manifestation, management, and pathogenesis. The steady rise in publications on VM/LM over the past 20 years, with a recent surge, reveals a growing global interest in VM/LM. The USA, Europe (Belgium and France), China, and Japan are the four leading study centers holding numerous leading scholars in VM/LM research. However, the collaboration and connection between different centers need to be strengthened. The current trend is gaining its ground, with primary interests on diagnosis, classification, genetic and targeted therapy, and sclerotherapy. Genetics-related pathogenesis and therapies have gradually gained focus in recent years. Future research should include molecular-level studies in diagnosis, classification, pathogenesis, precision targeted therapy, improvements to agents and methods in sclerotherapy, and AI application.

## Data Availability

All data and material used in this article can be retrieved from Web of Science. Detailed functions and parameters used in mentioned softwares are provided in Supplementary material.

## Footnotes

^1^ https://www.webofscience.com/wos/woscc/basic-search

^2^ https://www.bibliometrix.org
