# Supplementary material for: Global research trends and basis of venous/lymphatic malformations during 2003–2023: a bibliometric study over two decades
Source: Front Med (Lausanne). 2025 Apr 17;12:1555168. doi: 10.3389/fmed.2025.1555168 (PMC12045100; doi:10.3389/fmed.2025.1555168)
Supplement: Supplementary file 1 [file Table_1.DOCX]

Supplemantary Table 1: The Zone 1 journal based on the Bradford’s law

| **Journal Name** | **Rank** | **Freqency** | **cumulative Freqency** | **Zone** |
| --- | --- | --- | --- | --- |
| JOURNAL OF PEDIATRIC SURGERY | 1 | 92 | 92 | Zone 1 |
| JOURNAL OF CRANIOFACIAL SURGERY | 2 | 83 | 175 | Zone 1 |
| INTERNATIONAL JOURNAL OF PEDIATRIC OTORHINOLARYNGOLOGY | 3 | 77 | 252 | Zone 1 |
| PHLEBOLOGY | 4 | 70 | 322 | Zone 1 |
| PEDIATRIC DERMATOLOGY | 5 | 55 | 377 | Zone 1 |
| CUREUS JOURNAL OF MEDICAL SCIENCE | 6 | 54 | 431 | Zone 1 |
| INTERNATIONAL JOURNAL OF SURGERY CASE REPORTS | 7 | 48 | 479 | Zone 1 |
| JOURNAL OF PEDIATRIC SURGERY CASE REPORTS | 8 | 48 | 527 | Zone 1 |
| JOURNAL OF VASCULAR SURGERY-VENOUS AND LYMPHATIC DISORDERS | 9 | 47 | 574 | Zone 1 |
| OPHTHALMIC PLASTIC AND RECONSTRUCTIVE SURGERY | 10 | 47 | 621 | Zone 1 |
| PEDIATRIC RADIOLOGY | 11 | 47 | 668 | Zone 1 |
| MEDICINE | 12 | 43 | 711 | Zone 1 |
| PEDIATRIC BLOOD & CANCER | 13 | 42 | 753 | Zone 1 |
| DERMATOLOGIC SURGERY | 14 | 40 | 793 | Zone 1 |
| LARYNGOSCOPE | 15 | 38 | 831 | Zone 1 |
| LYMPHATIC RESEARCH AND BIOLOGY | 16 | 38 | 869 | Zone 1 |
| BMJ CASE REPORTS | 17 | 37 | 906 | Zone 1 |
| CARDIOVASCULAR AND INTERVENTIONAL RADIOLOGY | 18 | 37 | 943 | Zone 1 |
| JOURNAL OF VASCULAR AND INTERVENTIONAL RADIOLOGY | 19 | 37 | 980 | Zone 1 |
| AMERICAN JOURNAL OF ROENTGENOLOGY | 20 | 35 | 1015 | Zone 1 |
| PEDIATRIC SURGERY INTERNATIONAL | 21 | 35 | 1050 | Zone 1 |
| RADIOGRAPHICS | 22 | 31 | 1081 | Zone 1 |
| WORLD JOURNAL OF GASTROENTEROLOGY | 23 | 31 | 1112 | Zone 1 |
| OTOLARYNGOLOGY-HEAD AND NECK SURGERY | 24 | 29 | 1141 | Zone 1 |
| JOURNAL OF THE AMERICAN ACADEMY OF DERMATOLOGY | 25 | 28 | 1169 | Zone 1 |
| JOURNAL OF CUTANEOUS PATHOLOGY | 26 | 27 | 1196 | Zone 1 |
| JOURNAL OF VASCULAR SURGERY | 27 | 26 | 1222 | Zone 1 |
| EUROPEAN JOURNAL OF PEDIATRIC SURGERY | 28 | 25 | 1247 | Zone 1 |
| JOURNAL OF ORAL AND MAXILLOFACIAL SURGERY | 29 | 25 | 1272 | Zone 1 |
| LYMPHOLOGY | 30 | 25 | 1297 | Zone 1 |
| JOURNAL OF PLASTIC RECONSTRUCTIVE AND AESTHETIC SURGERY | 31 | 24 | 1321 | Zone 1 |
| FRONTIERS IN PEDIATRICS | 32 | 23 | 1344 | Zone 1 |
| JOURNAL OF ULTRASOUND IN MEDICINE | 33 | 22 | 1366 | Zone 1 |
| PEDIATRICS | 34 | 22 | 1388 | Zone 1 |
| WORLD JOURNAL OF CLINICAL CASES | 35 | 22 | 1410 | Zone 1 |
| ANNALS OF MEDICINE AND SURGERY | 36 | 21 | 1431 | Zone 1 |
| BRITISH JOURNAL OF ORAL & MAXILLOFACIAL SURGERY | 37 | 20 | 1451 | Zone 1 |
| JOURNAL OF LARYNGOLOGY AND OTOLOGY | 38 | 20 | 1471 | Zone 1 |
| ORPHANET JOURNAL OF RARE DISEASES | 39 | 20 | 1491 | Zone 1 |
| SURGERY TODAY | 40 | 20 | 1511 | Zone 1 |
| ANNALS OF VASCULAR SURGERY | 41 | 19 | 1530 | Zone 1 |
| AMERICAN SURGEON | 42 | 18 | 1548 | Zone 1 |
| ANNALS OF PLASTIC SURGERY | 43 | 18 | 1566 | Zone 1 |
| CLINICAL IMAGING | 44 | 18 | 1584 | Zone 1 |
| CLINICAL RADIOLOGY | 45 | 18 | 1602 | Zone 1 |
| EUROPEAN ARCHIVES OF OTO-RHINO-LARYNGOLOGY | 46 | 18 | 1620 | Zone 1 |
| EUROPEAN JOURNAL OF RADIOLOGY | 47 | 18 | 1638 | Zone 1 |
| EUROPEAN RADIOLOGY | 48 | 18 | 1656 | Zone 1 |
