# Supplementary material for: Global research trends and basis of venous/lymphatic malformations during 2003–2023: a bibliometric study over two decades
Source: Front Med (Lausanne). 2025 Apr 17;12:1555168. doi: 10.3389/fmed.2025.1555168 (PMC12045100; doi:10.3389/fmed.2025.1555168)
Supplement: Supplementary file 2 [file Supplementary_file_1.docx]

**Supplementary File**

The exact functions and parameters used in VOSviewer, CiteSpace and R package “bibliometrix” are provided as follows in order of figures:

**Figure 3C:**

VOSviewer: institutions with the number of publications less than 5 were excluded.

**Figure 4:**

**A.** VOSviewer: authors with the number of publications less than 5 were excluded. Articles with more than 25 authors had been automatically excluded.

**B.** VOSviewer: authors with the number of co-citations less than 40 were excluded.

**Supplementary Figure 3:**

VOSviewer: journals with the number of publications less than 5 were excluded.

**Figure 5E:**

VOSviewer: keywords with the number of occurrences less than 5 were excluded.

**Supplementary Figure 4:**

VOSviewer: references with the number of co-citations less than 20 were excluded.

**Supplementary Figure 1 A&B:**

VOSviewer: countries with the number of publications less than 5 were excluded.

CiteSpace:

1. Settings: Term Type -> Burst Terms; Node Types -> Keyword.

2. GO -> Visualize.

3. Control Panel -> Burstness -> View.

R package “bibliometrix”: Words -> Trend Topics.
