# Supplementary figures and images for: Global research trends and basis of venous/lymphatic malformations during 2003–2023: a bibliometric study over two decades
Source: Front Med (Lausanne). 2025 Apr 17;12:1555168. doi: 10.3389/fmed.2025.1555168 (PMC12045100; doi:10.3389/fmed.2025.1555168)

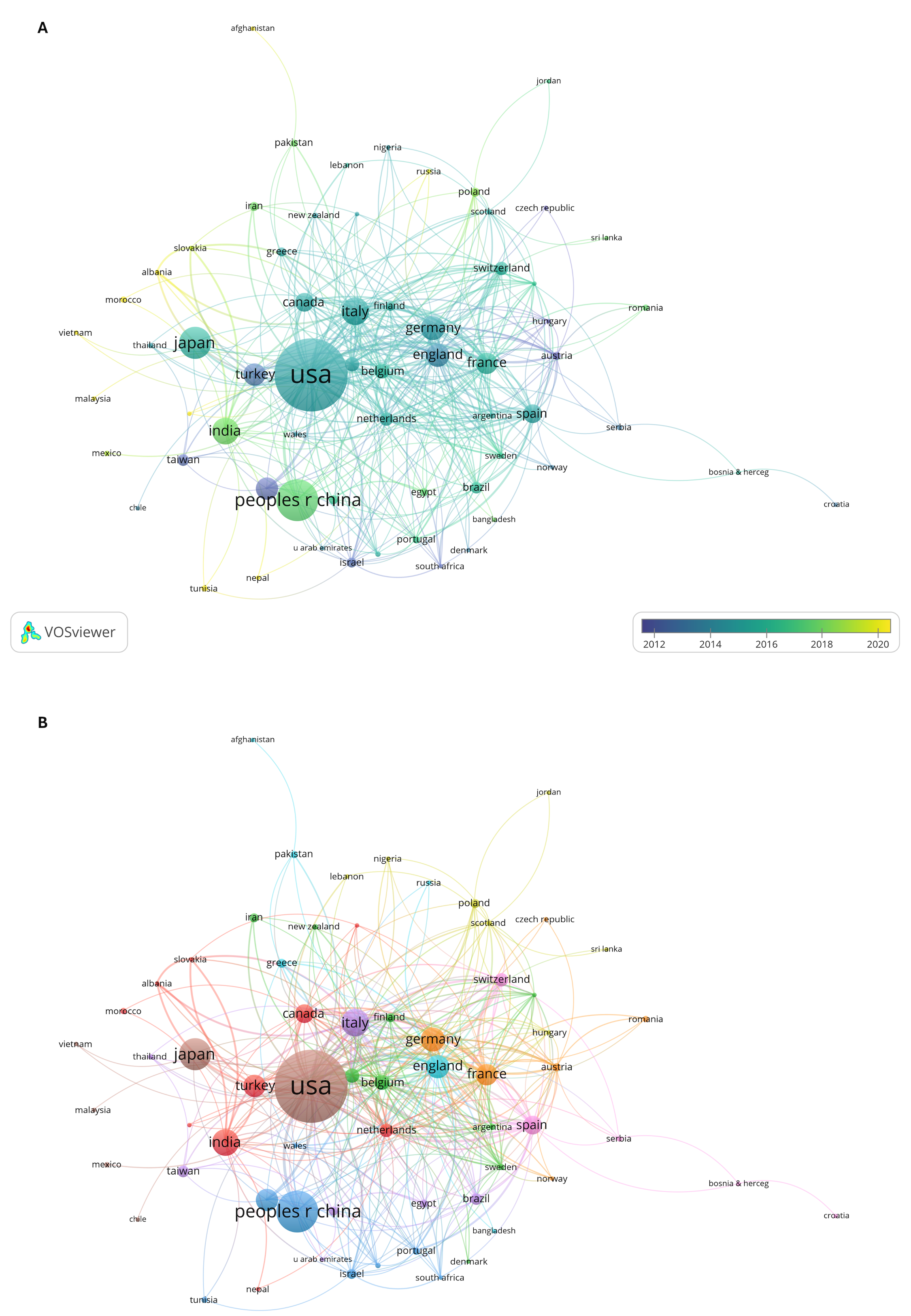

Supplement: Supplementary file 3 [file Image_1.TIF]

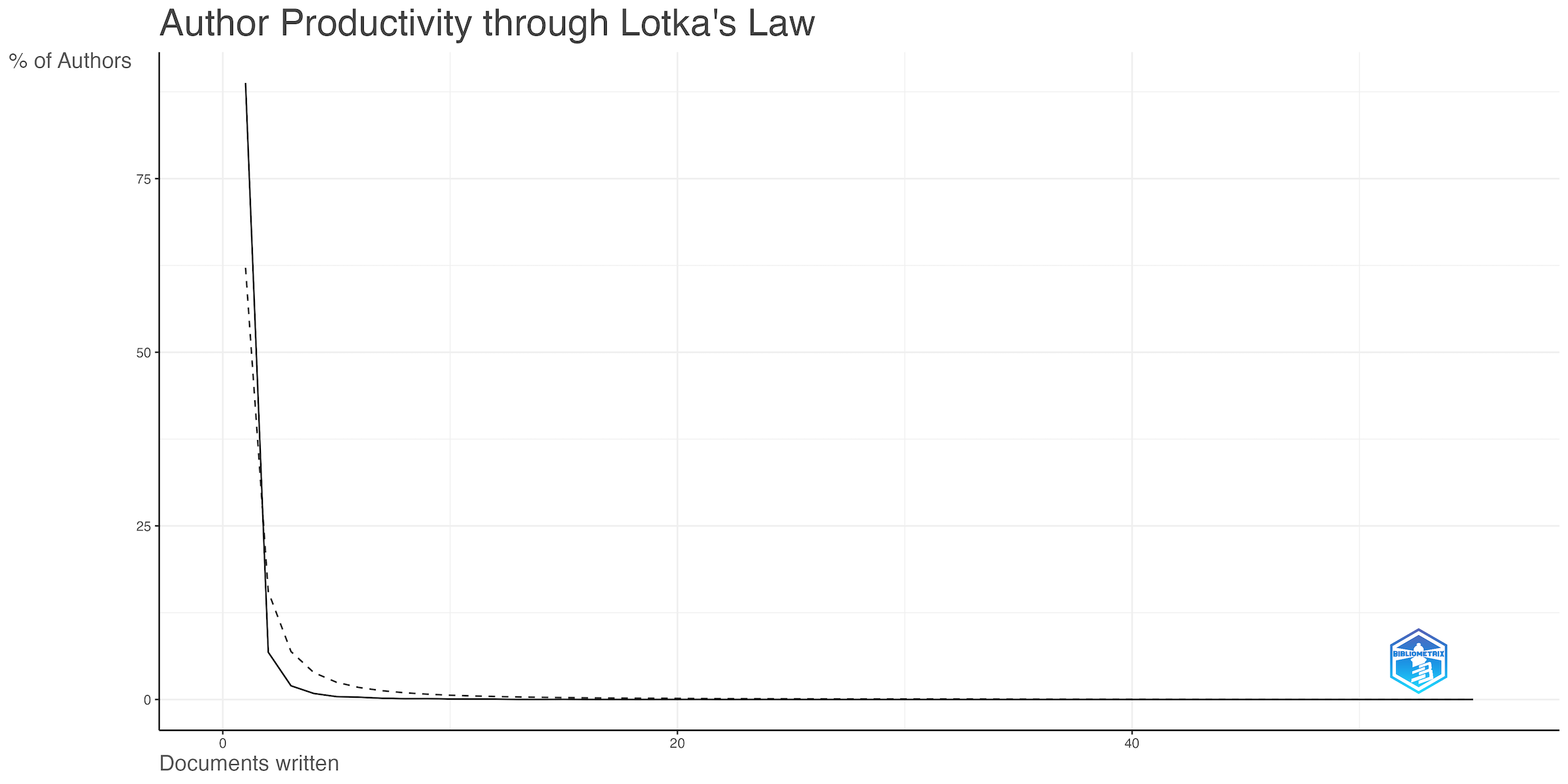

Supplement: Supplementary file 4 [file Image_2.TIF]

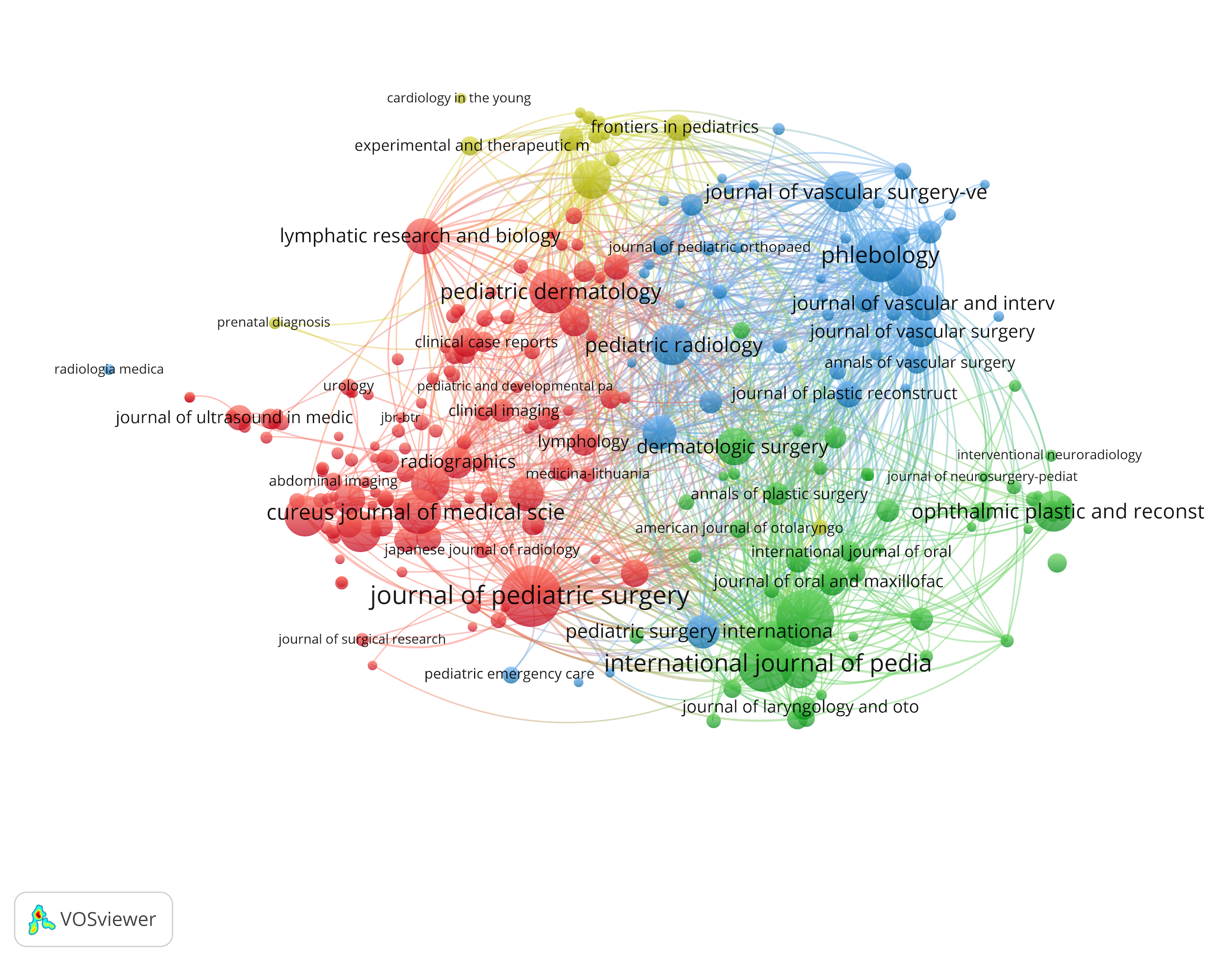

Supplement: Supplementary file 5 [file Image_3.TIF]

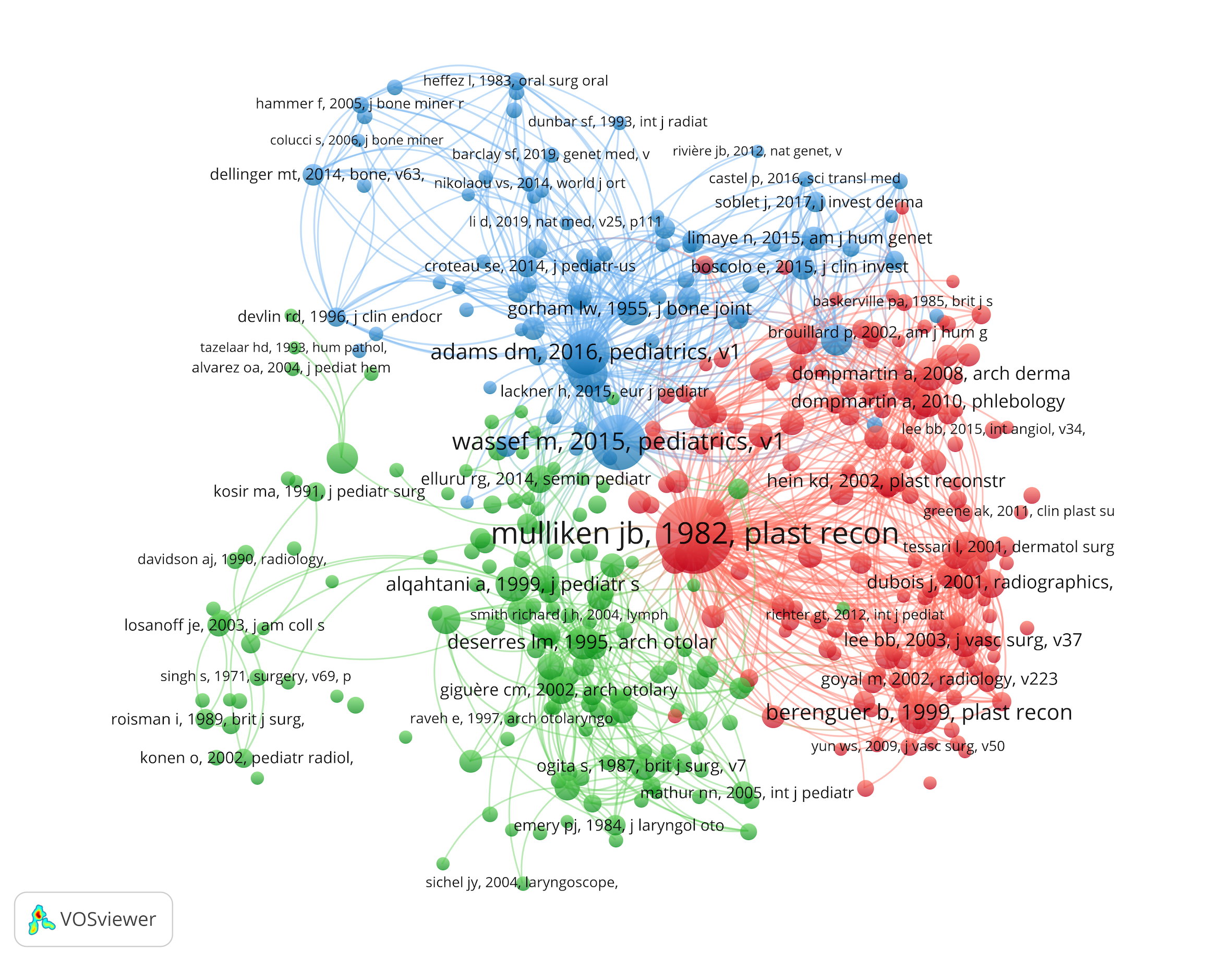

Supplement: Supplementary file 6 [file Image_4.TIF]
